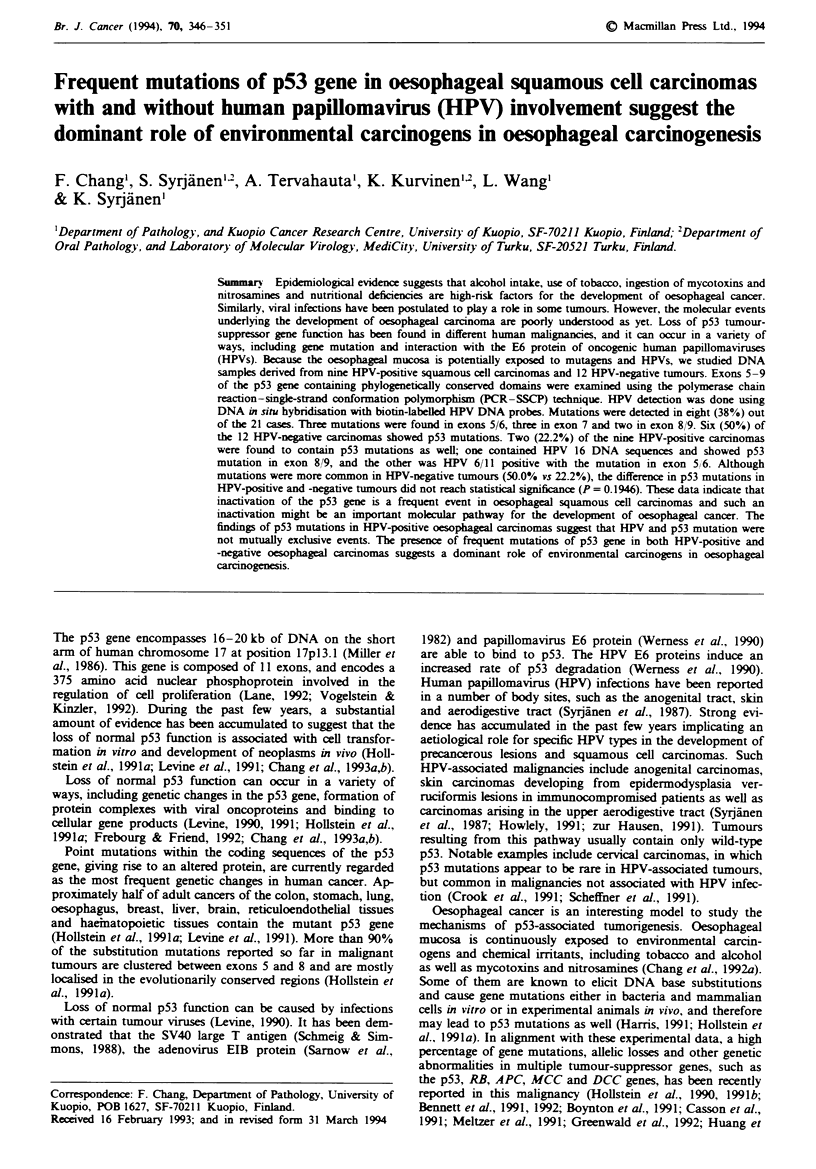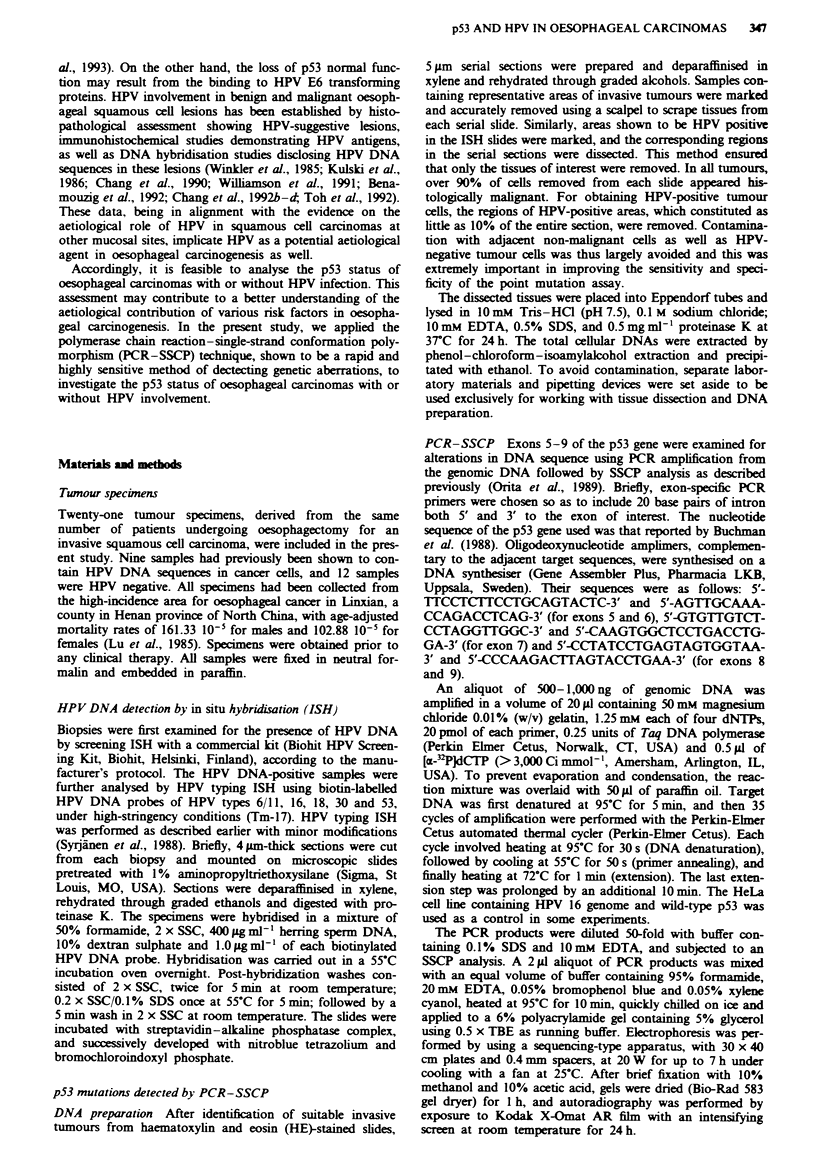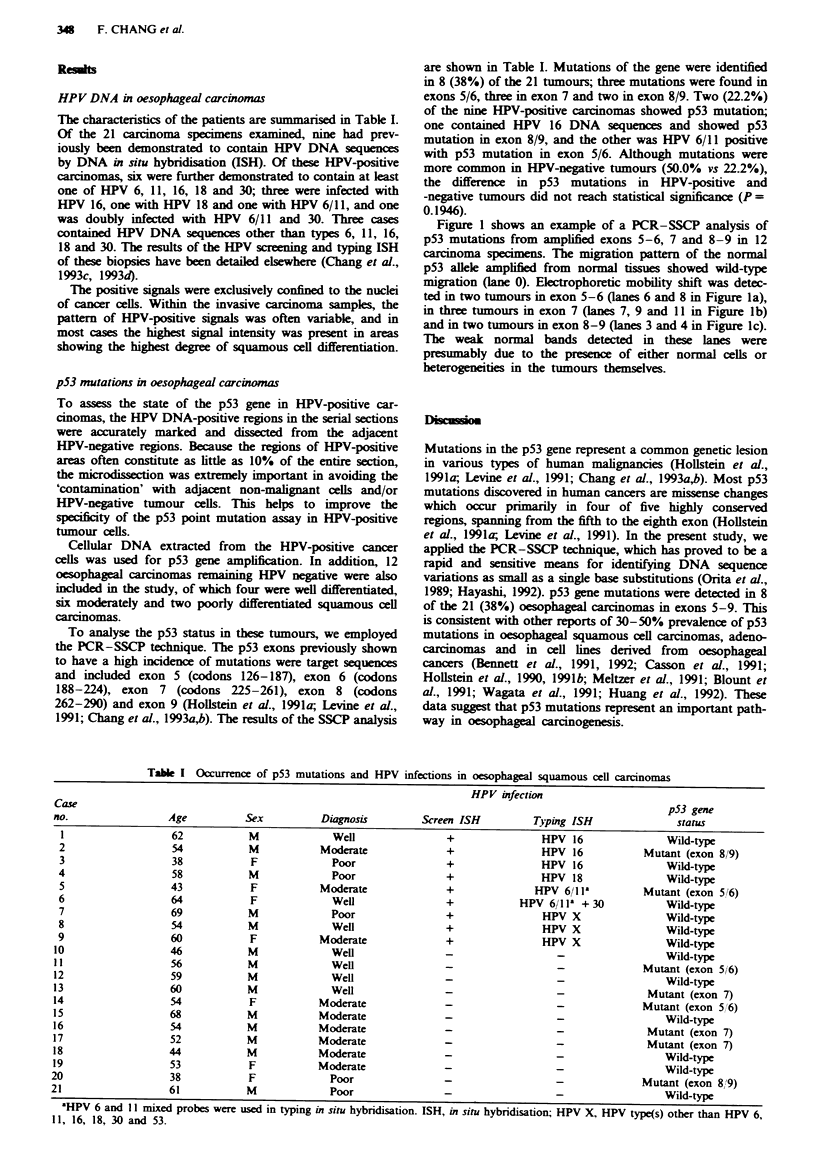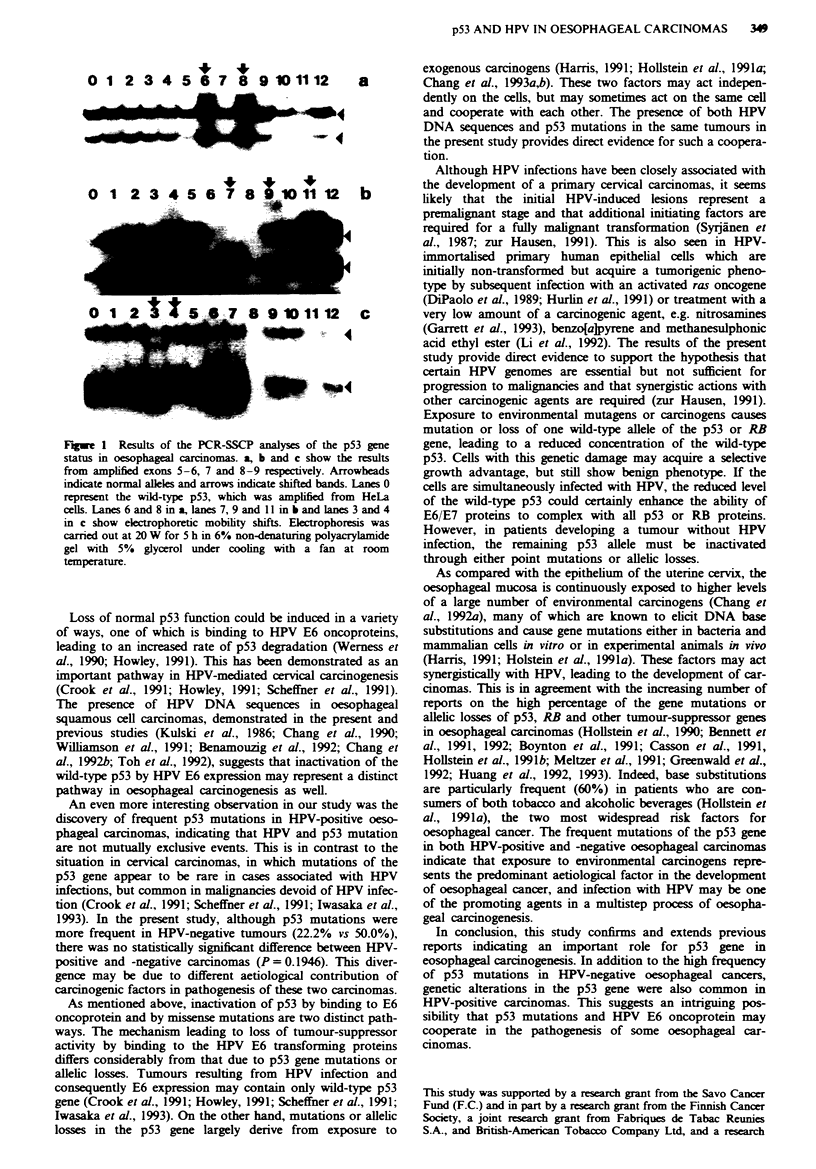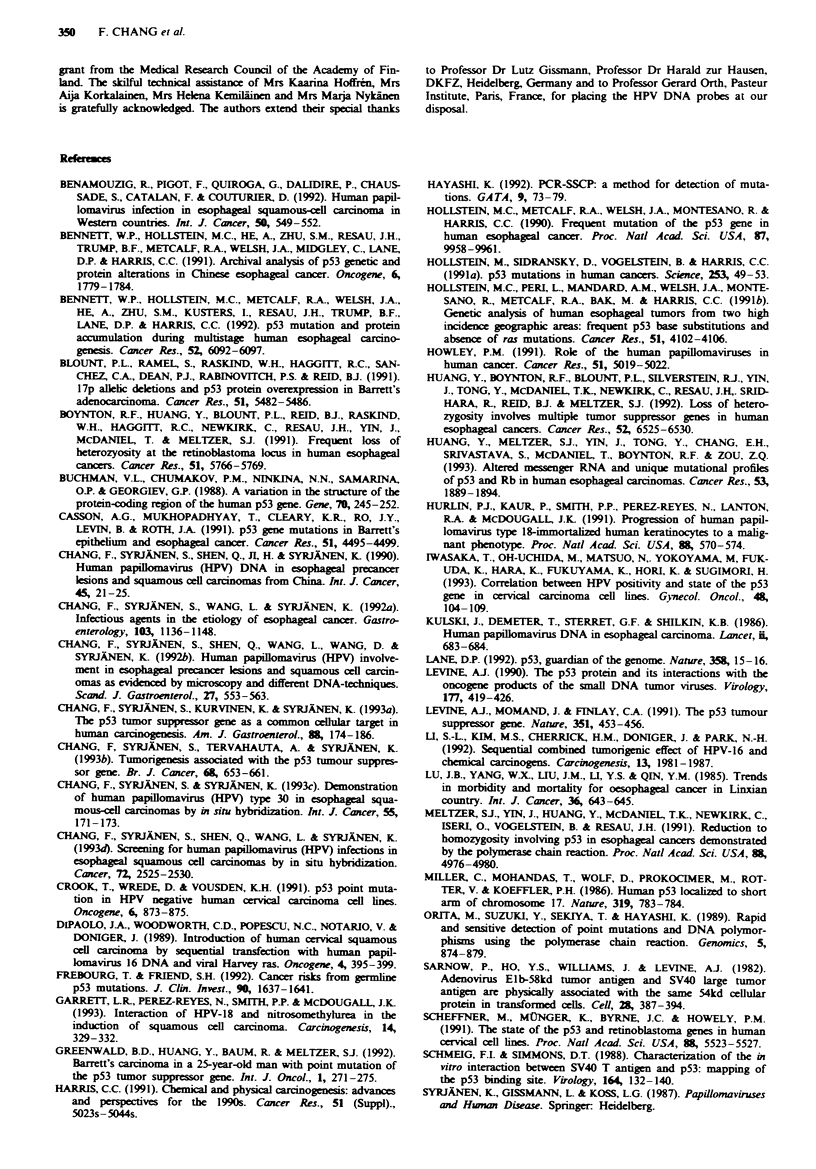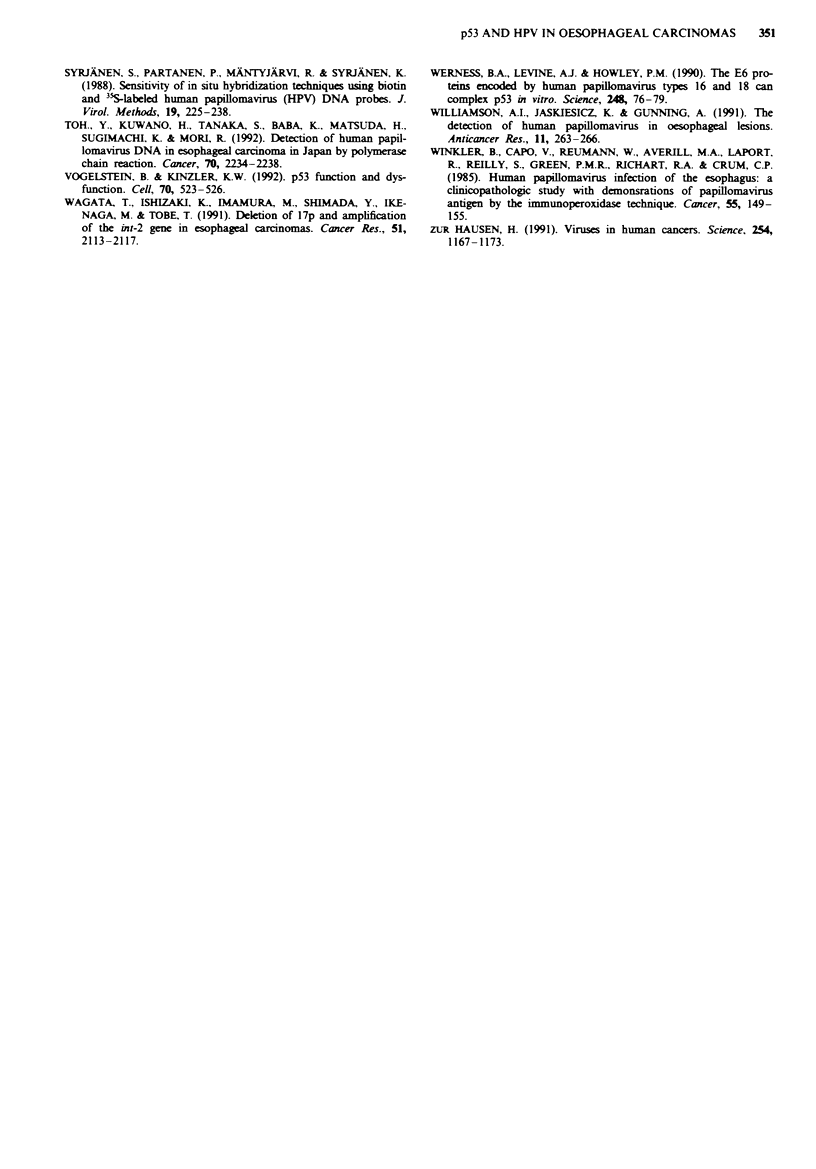# Frequent mutations of p53 gene in oesophageal squamous cell carcinomas with and without human papillomavirus (HPV) involvement suggest the dominant role of environmental carcinogens in oesophageal carcinogenesis.

**DOI:** 10.1038/bjc.1994.305

**Published:** 1994-08

**Authors:** F. Chang, S. Syrjänen, A. Tervahauta, K. Kurvinen, L. Wang, K. Syrjänen

**Affiliations:** Department of Pathology, University of Kuopio, Finland.

## Abstract

**Images:**


Br. J. Cancer (1994), 70, 346-351 © Macmillan Press Ltd., 1994

### Frequent mutations of p53 gene in oesophageal squamous cell carcinomas with and without human papillomavirus (HPV) involvement suggest the dominant role of environmental carcinogens in oesophageal carcinogenesis

F. Chang[1], S. Syrjänen[1,2], A. Tervahauta[1], K. Kurvinen[1,2], L. Wang[1] & K. Syrjänen[1]

[1]*Department of Pathology, and Kuopio Cancer Research Centre, University of Kuopio, SF-70211 Kuopio, Finland;* [2]*Department of Oral Pathology, and Laboratory of Molecular Virology, MediCity, University of Turku, SF-20521 Turku, Finland.*

**Summary** Epidemiological evidence suggests that alcohol intake, use of tobacco, ingestion of mycotoxins and nitrosamines and nutritional deficiencies are high-risk factors for the development of oesophageal cancer. Similarly, viral infections have been postulated to play a role in some tumours. However, the molecular events underlying the development of oesophageal carcinoma are poorly understood as yet. Loss of p53 tumour-suppressor gene function has been found in different human malignancies, and it can occur in a variety of ways, including gene mutation and interaction with the E6 protein of oncogenic human papillomaviruses (HPVs). Because the oesophageal mucosa is potentially exposed to mutagens and HPVs, we studied DNA samples derived from nine HPV-positive squamous cell carcinomas and 12 HPV-negative tumours. Exons 5-9 of the p53 gene containing phylogenetically conserved domains were examined using the polymerase chain reaction-single-strand conformation polymorphism (PCR-SSCP) technique. HPV detection was done using DNA *in situ* hybridisation with biotin-labelled HPV DNA probes. Mutations were detected in eight (38%) out of the 21 cases. Three mutations were found in exons 5/6, three in exon 7 and two in exon 8/9. Six (50%) of the 12 HPV-negative carcinomas showed p53 mutations. Two (22.2%) of the nine HPV-positive carcinomas were found to contain p53 mutations as well; one contained HPV 16 DNA sequences and showed p53 mutation in exon 8/9, and the other was HPV 6/11 positive with the mutation in exon 5/6. Although mutations were more common in HPV-negative tumours (50.0% *vs* 22.2%), the difference in p53 mutations in HPV-positive and -negative tumours did not reach statistical significance ($P = 0.1946$). These data indicate that inactivation of the p53 gene is a frequent event in oesophageal squamous cell carcinomas and such an inactivation might be an important molecular pathway for the development of oesophageal cancer. The findings of p53 mutations in HPV-positive oesophageal carcinomas suggest that HPV and p53 mutation were not mutually exclusive events. The presence of frequent mutations of p53 gene in both HPV-positive and -negative oesophageal carcinomas suggests a dominant role of environmental carcinogens in oesophageal carcinogenesis.

The p53 gene encompasses 16-20 kb of DNA on the short arm of human chromosome 17 at position 17p13.1 (Miller *et al.*, 1986). This gene is composed of 11 exons, and encodes a 375 amino acid nuclear phosphoprotein involved in the regulation of cell proliferation (Lane, 1992; Vogelstein & Kinzler, 1992). During the past few years, a substantial amount of evidence has been accumulated to suggest that the loss of normal p53 function is associated with cell transformation *in vitro* and development of neoplasms *in vivo* (Hollstein *et al.*, 1991*a*; Levine *et al.*, 1991; Chang *et al.*, 1993*a*,*b*).

Loss of normal p53 function can occur in a variety of ways, including genetic changes in the p53 gene, formation of protein complexes with viral oncoproteins and binding to cellular gene products (Levine, 1990, 1991; Hollstein *et al.*, 1991*a*; Frebourg & Friend, 1992; Chang *et al.*, 1993*a*,*b*).

Point mutations within the coding sequences of the p53 gene, giving rise to an altered protein, are currently regarded as the most frequent genetic changes in human cancer. Approximately half of adult cancers of the colon, stomach, lung, oesophagus, breast, liver, brain, reticuloendothelial tissues and haematopoietic tissues contain the mutant p53 gene (Hollstein *et al.*, 1991*a*; Levine *et al.*, 1991). More than 90% of the substitution mutations reported so far in malignant tumours are clustered between exons 5 and 8 and are mostly localised in the evolutionarily conserved regions (Hollstein *et al.*, 1991*a*).

Loss of normal p53 function can be caused by infections with certain tumour viruses (Levine, 1990). It has been demonstrated that the SV40 large T antigen (Schmeig & Simmons, 1988), the adenovirus EIB protein (Sarnow *et al.*, 1982) and papillomavirus E6 protein (Werness *et al.*, 1990) are able to bind to p53. The HPV E6 proteins induce an increased rate of p53 degradation (Werness *et al.*, 1990). Human papillomavirus (HPV) infections have been reported in a number of body sites, such as the anogenital tract, skin and aerodigestive tract (Syrjänen *et al.*, 1987). Strong evidence has accumulated in the past few years implicating an aetiological role for specific HPV types in the development of precancerous lesions and squamous cell carcinomas. Such HPV-associated malignancies include anogenital carcinomas, skin carcinomas developing from epidermodysplasia verruciformis lesions in immunocompromised patients as well as carcinomas arising in the upper aerodigestive tract (Syrjänen *et al.*, 1987; Howlely, 1991; zur Hausen, 1991). Tumours resulting from this pathway usually contain only wild-type p53. Notable examples include cervical carcinomas, in which p53 mutations appear to be rare in HPV-associated tumours, but common in malignancies not associated with HPV infection (Crook *et al.*, 1991; Scheffner *et al.*, 1991).

Oesophageal cancer is an interesting model to study the mechanisms of p53-associated tumorigenesis. Oesophageal mucosa is continuously exposed to environmental carcinogens and chemical irritants, including tobacco and alcohol as well as mycotoxins and nitrosamines (Chang *et al.*, 1992*a*). Some of them are known to elicit DNA base substitutions and cause gene mutations either in bacteria and mammalian cells *in vitro* or in experimental animals *in vivo*, and therefore may lead to p53 mutations as well (Harris, 1991; Hollstein *et al.*, 1991*a*). In alignment with these experimental data, a high percentage of gene mutations, allelic losses and other genetic abnormalities in multiple tumour-suppressor genes, such as the p53, *RB*, *APC*, *MCC* and *DCC* genes, has been recently reported in this malignancy (Hollstein *et al.*, 1990, 1991*b*; Bennett *et al.*, 1991, 1992; Boynton *et al.*, 1991; Casson *et al.*, 1991; Meltzer *et al.*, 1991; Greenwald *et al.*, 1992; Huang *et*

Correspondence: F. Chang, Department of Pathology, University of Kuopio, POB 1627, SF-70211 Kuopio, Finland.
Received 16 February 1993; and in revised form 31 March 1994

al., 1993). On the other hand, the loss of p53 normal function may result from the binding to HPV E6 transforming proteins. HPV involvement in benign and malignant oesophageal squamous cell lesions has been established by histopathological assessment showing HPV-suggestive lesions, immunohistochemical studies demonstrating HPV antigens, as well as DNA hybridisation studies disclosing HPV DNA sequences in these lesions (Winkler *et al.*, 1985; Kulski *et al.*, 1986; Chang *et al.*, 1990; Williamson *et al.*, 1991; Benamouzig *et al.*, 1992; Chang *et al.*, 1992*b*–*d*; Toh *et al.*, 1992). These data, being in alignment with the evidence on the aetiological role of HPV in squamous cell carcinomas at other mucosal sites, implicate HPV as a potential aetiological agent in oesophageal carcinogenesis as well.

Accordingly, it is feasible to analyse the p53 status of oesophageal carcinomas with or without HPV infection. This assessment may contribute to a better understanding of the aetiological contribution of various risk factors in oesophageal carcinogenesis. In the present study, we applied the polymerase chain reaction–single-strand conformation polymorphism (PCR–SSCP) technique, shown to be a rapid and highly sensitive method of dectecting genetic aberrations, to investigate the p53 status of oesophageal carcinomas with or without HPV involvement.

### Materials and methods

### *Tumour specimens*

Twenty-one tumour specimens, derived from the same number of patients undergoing oesophagectomy for an invasive squamous cell carcinoma, were included in the present study. Nine samples had previously been shown to contain HPV DNA sequences in cancer cells, and 12 samples were HPV negative. All specimens had been collected from the high-incidence area for oesophageal cancer in Linxian, a county in Henan province of North China, with age-adjusted mortality rates of 161.33 $10^{-5}$ for males and 102.88 $10^{-5}$ for females (Lu *et al.*, 1985). Specimens were obtained prior to any clinical therapy. All samples were fixed in neutral formalin and embedded in paraffin.

### *HPV DNA detection by* in situ *hybridisation (ISH)*

Biopsies were first examined for the presence of HPV DNA by screening ISH with a commercial kit (Biohit HPV Screening Kit, Biohit, Helsinki, Finland), according to the manufacturer's protocol. The HPV DNA-positive samples were further analysed by HPV typing ISH using biotin-labelled HPV DNA probes of HPV types 6/11, 16, 18, 30 and 53, under high-stringency conditions (Tm-17). HPV typing ISH was performed as described earlier with minor modifications (Syrjänen *et al.*, 1988). Briefly, 4 µm-thick sections were cut from each biopsy and mounted on microscopic slides pretreated with 1% aminopropyltriethoxysilane (Sigma, St Louis, MO, USA). Sections were deparaffinised in xylene, rehydrated through graded ethanols and digested with proteinase K. The specimens were hybridised in a mixture of 50% formamide, 2 × SSC, 400 µg $ml^{-1}$ herring sperm DNA, 10% dextran sulphate and 1.0 µg $ml^{-1}$ of each biotinylated HPV DNA probe. Hybridisation was carried out in a 55°C incubation oven overnight. Post-hybridization washes consisted of 2 × SSC, twice for 5 min at room temperature; 0.2 × SSC/0.1% SDS once at 55°C for 5 min; followed by a 5 min wash in 2 × SSC at room temperature. The slides were incubated with streptavidin–alkaline phosphatase complex, and successively developed with nitroblue tetrazolium and bromochloroindoxyl phosphate.

### *p53 mutations detected by PCR–SSCP*

*DNA preparation* After identification of suitable invasive tumours from haematoxylin and eosin (HE)-stained slides, 5 µm serial sections were prepared and deparaffinised in xylene and rehydrated through graded alcohols. Samples containing representative areas of invasive tumours were marked and accurately removed using a scalpel to scrape tissues from each serial slide. Similarly, areas shown to be HPV positive in the ISH slides were marked, and the corresponding regions in the serial sections were dissected. This method ensured that only the tissues of interest were removed. In all tumours, over 90% of cells removed from each slide appeared histologically malignant. For obtaining HPV-positive tumour cells, the regions of HPV-positive areas, which constituted as little as 10% of the entire section, were removed. Contamination with adjacent non-malignant cells as well as HPV-negative tumour cells was thus largely avoided and this was extremely important in improving the sensitivity and specificity of the point mutation assay.

The dissected tissues were placed into Eppendorf tubes and lysed in 10 mM Tris–HCl (pH 7.5), 0.1 M sodium chloride; 10 mM EDTA, 0.5% SDS, and 0.5 mg $ml^{-1}$ proteinase K at 37°C for 24 h. The total cellular DNAs were extracted by phenol–chloroform–isoamylalcohol extraction and precipitated with ethanol. To avoid contamination, separate laboratory materials and pipetting devices were set aside to be used exclusively for working with tissue dissection and DNA preparation.

*PCR–SSCP* Exons 5–9 of the p53 gene were examined for alterations in DNA sequence using PCR amplification from the genomic DNA followed by SSCP analysis as described previously (Orita *et al.*, 1989). Briefly, exon-specific PCR primers were chosen so as to include 20 base pairs of intron both 5′ and 3′ to the exon of interest. The nucleotide sequence of the p53 gene used was that reported by Buchman *et al.* (1988). Oligodeoxynucleotide amplimers, complementary to the adjacent target sequences, were synthesised on a DNA synthesiser (Gene Assembler Plus, Pharmacia LKB, Uppsala, Sweden). Their sequences were as follows: 5′-TTCCTCTTCCTGCAGTACTC-3′ and 5′-AGTTGCAAACCAGACCTCAG-3′ (for exons 5 and 6), 5′-GTGTTGTCTCCTAGGTTGGC-3′ and 5′-CAAGTGGCTCCTGACCTGGA-3′ (for exon 7) and 5′-CCTATCCTGAGTAGTGGTAA-3′ and 5′-CCCAAGACTTAGTACCTGAA-3′ (for exons 8 and 9).

An aliquot of 500–1,000 ng of genomic DNA was amplified in a volume of 20 µl containing 50 mM magnesium chloride 0.01% (w/v) gelatin, 1.25 mM each of four dNTPs, 20 pmol of each primer, 0.25 units of *Taq* DNA polymerase (Perkin Elmer Cetus, Norwalk, CT, USA) and 0.5 µl of [α-$^{32}$P]dCTP (> 3,000 Ci $mmol^{-1}$, Amersham, Arlington, IL, USA). To prevent evaporation and condensation, the reaction mixture was overlaid with 50 µl of paraffin oil. Target DNA was first denatured at 95°C for 5 min, and then 35 cycles of amplification were performed with the Perkin-Elmer Cetus automated thermal cycler (Perkin-Elmer Cetus). Each cycle involved heating at 95°C for 30 s (DNA denaturation), followed by cooling at 55°C for 50 s (primer annealing), and finally heating at 72°C for 1 min (extension). The last extension step was prolonged by an additional 10 min. The HeLa cell line containing HPV 16 genome and wild-type p53 was used as a control in some experiments.

The PCR products were diluted 50-fold with buffer containing 0.1% SDS and 10 mM EDTA, and subjected to an SSCP analysis. A 2 µl aliquot of PCR products was mixed with an equal volume of buffer containing 95% formamide, 20 mM EDTA, 0.05% bromophenol blue and 0.05% xylene cyanol, heated at 95°C for 10 min, quickly chilled on ice and applied to a 6% polyacrylamide gel containing 5% glycerol using 0.5 × TBE as running buffer. Electrophoresis was performed by using a sequencing-type apparatus, with 30 × 40 cm plates and 0.4 mm spacers, at 20 W for up to 7 h under cooling with a fan at 25°C. After brief fixation with 10% methanol and 10% acetic acid, gels were dried (Bio-Rad 583 gel dryer) for 1 h, and autoradiography was performed by exposure to Kodak X-Omat AR film with an intensifying screen at room temperature for 24 h.

### Results

### HPV DNA in oesophageal carcinomas

The characteristics of the patients are summarised in Table I. Of the 21 carcinoma specimens examined, nine had previously been demonstrated to contain HPV DNA sequences by DNA *in situ* hybridisation (ISH). Of these HPV-positive carcinomas, six were further demonstrated to contain at least one of HPV 6, 11, 16, 18 and 30; three were infected with HPV 16, one with HPV 18 and one with HPV 6/11, and one was doubly infected with HPV 6/11 and 30. Three cases contained HPV DNA sequences other than types 6, 11, 16, 18 and 30. The results of the HPV screening and typing ISH of these biopsies have been detailed elsewhere (Chang *et al.*, 1993*c*, 1993*d*).

The positive signals were exclusively confined to the nuclei of cancer cells. Within the invasive carcinoma samples, the pattern of HPV-positive signals was often variable, and in most cases the highest signal intensity was present in areas showing the highest degree of squamous cell differentiation.

### p53 mutations in oesophageal carcinomas

To assess the state of the p53 gene in HPV-positive carcinomas, the HPV DNA-positive regions in the serial sections were accurately marked and dissected from the adjacent HPV-negative regions. Because the regions of HPV-positive areas often constitute as little as 10% of the entire section, the microdissection was extremely important in avoiding the 'contamination' with adjacent non-malignant cells and/or HPV-negative tumour cells. This helps to improve the specificity of the p53 point mutation assay in HPV-positive tumour cells.

Cellular DNA extracted from the HPV-positive cancer cells was used for p53 gene amplification. In addition, 12 oesophageal carcinomas remaining HPV negative were also included in the study, of which four were well differentiated, six moderately and two poorly differentiated squamous cell carcinomas.

To analyse the p53 status in these tumours, we employed the PCR-SSCP technique. The p53 exons previously shown to have a high incidence of mutations were target sequences and included exon 5 (codons 126-187), exon 6 (codons 188-224), exon 7 (codons 225-261), exon 8 (codons 262-290) and exon 9 (Hollstein *et al.*, 1991*a*; Levine *et al.*, 1991; Chang *et al.*, 1993*a*,*b*). The results of the SSCP analysis are shown in Table I. Mutations of the gene were identified in 8 (38%) of the 21 tumours; three mutations were found in exons 5/6, three in exon 7 and two in exon 8/9. Two (22.2%) of the nine HPV-positive carcinomas showed p53 mutation; one contained HPV 16 DNA sequences and showed p53 mutation in exon 8/9, and the other was HPV 6/11 positive with p53 mutation in exon 5/6. Although mutations were more common in HPV-negative tumours (50.0% *vs* 22.2%), the difference in p53 mutations in HPV-positive and -negative tumours did not reach statistical significance ($P = 0.1946$).

Figure 1 shows an example of a PCR-SSCP analysis of p53 mutations from amplified exons 5-6, 7 and 8-9 in 12 carcinoma specimens. The migration pattern of the normal p53 allele amplified from normal tissues showed wild-type migration (lane 0). Electrophoretic mobility shift was detected in two tumours in exon 5-6 (lanes 6 and 8 in Figure 1a), in three tumours in exon 7 (lanes 7, 9 and 11 in Figure 1b) and in two tumours in exon 8-9 (lanes 3 and 4 in Figure 1c). The weak normal bands detected in these lanes were presumably due to the presence of either normal cells or heterogeneities in the tumours themselves.

### Discussion

Mutations in the p53 gene represent a common genetic lesion in various types of human malignancies (Hollstein *et al.*, 1991*a*; Levine *et al.*, 1991; Chang *et al.*, 1993*a*,*b*). Most p53 mutations discovered in human cancers are missense changes which occur primarily in four of five highly conserved regions, spanning from the fifth to the eighth exon (Hollstein *et al.*, 1991*a*; Levine *et al.*, 1991). In the present study, we applied the PCR-SSCP technique, which has proved to be a rapid and sensitive means for identifying DNA sequence variations as small as a single base substitutions (Orita *et al.*, 1989; Hayashi, 1992). p53 gene mutations were detected in 8 of the 21 (38%) oesophageal carcinomas in exons 5-9. This is consistent with other reports of 30-50% prevalence of p53 mutations in oesophageal squamous cell carcinomas, adenocarcinomas and in cell lines derived from oesophageal cancers (Bennett *et al.*, 1991, 1992; Casson *et al.*, 1991; Hollstein *et al.*, 1990, 1991*b*; Meltzer *et al.*, 1991; Blount *et al.*, 1991; Wagata *et al.*, 1991; Huang *et al.*, 1992). These data suggest that p53 mutations represent an important pathway in oesophageal carcinogenesis.

**Table I** Occurrence of p53 mutations and HPV infections in oesophageal squamous cell carcinomas

| Case no. | Age | Sex | Diagnosis | HPV infection: Screen ISH | HPV infection: Typing ISH | p53 gene status |
|---|---|---|---|---|---|---|
| 1 | 62 | M | Well | + | HPV 16 | Wild-type |
| 2 | 54 | M | Moderate | + | HPV 16 | Mutant (exon 8/9) |
| 3 | 38 | F | Poor | + | HPV 16 | Wild-type |
| 4 | 58 | M | Poor | + | HPV 18 | Wild-type |
| 5 | 43 | F | Moderate | + | HPV 6/11[a] | Mutant (exon 5/6) |
| 6 | 64 | F | Well | + | HPV 6/11[a] + 30 | Wild-type |
| 7 | 69 | M | Poor | + | HPV X | Wild-type |
| 8 | 54 | M | Well | + | HPV X | Wild-type |
| 9 | 60 | F | Moderate | + | HPV X | Wild-type |
| 10 | 46 | M | Well | – | – | Wild-type |
| 11 | 56 | M | Well | – | – | Mutant (exon 5/6) |
| 12 | 59 | M | Well | – | – | Wild-type |
| 13 | 60 | M | Well | – | – | Mutant (exon 7) |
| 14 | 54 | F | Moderate | – | – | Mutant (exon 5/6) |
| 15 | 68 | M | Moderate | – | – | Wild-type |
| 16 | 54 | M | Moderate | – | – | Mutant (exon 7) |
| 17 | 52 | M | Moderate | – | – | Mutant (exon 7) |
| 18 | 44 | M | Moderate | – | – | Wild-type |
| 19 | 53 | F | Moderate | – | – | Wild-type |
| 20 | 38 | F | Poor | – | – | Mutant (exon 8/9) |
| 21 | 61 | M | Poor | – | – | Wild-type |

[a]HPV 6 and 11 mixed probes were used in typing *in situ* hybridisation. ISH, *in situ* hybridisation; HPV X, HPV type(s) other than HPV 6, 11, 16, 18, 30 and 53.

**Figure 1** Results of the PCR-SSCP analyses of the p53 gene status in oesophageal carcinomas. **a**, **b** and **c** show the results from amplified exons 5–6, 7 and 8–9 respectively. Arrowheads indicate normal alleles and arrows indicate shifted bands. Lanes 0 represent the wild-type p53, which was amplified from HeLa cells. Lanes 6 and 8 in **a**, lanes 7, 9 and 11 in **b** and lanes 3 and 4 in **c** show electrophoretic mobility shifts. Electrophoresis was carried out at 20 W for 5 h in 6% non-denaturing polyacrylamide gel with 5% glycerol under cooling with a fan at room temperature.

Loss of normal p53 function could be induced in a variety of ways, one of which is binding to HPV E6 oncoproteins, leading to an increased rate of p53 degradation (Werness *et al.*, 1990; Howley, 1991). This has been demonstrated as an important pathway in HPV-mediated cervical carcinogenesis (Crook *et al.*, 1991; Howley, 1991; Scheffner *et al.*, 1991). The presence of HPV DNA sequences in oesophageal squamous cell carcinomas, demonstrated in the present and previous studies (Kulski *et al.*, 1986; Chang *et al.*, 1990; Williamson *et al.*, 1991; Benamouzig *et al.*, 1992; Chang *et al.*, 1992*b*; Toh *et al.*, 1992), suggests that inactivation of the wild-type p53 by HPV E6 expression may represent a distinct pathway in oesophageal carcinogenesis as well.

An even more interesting observation in our study was the discovery of frequent p53 mutations in HPV-positive oesophageal carcinomas, indicating that HPV and p53 mutation are not mutually exclusive events. This is in contrast to the situation in cervical carcinomas, in which mutations of the p53 gene appear to be rare in cases associated with HPV infections, but common in malignancies devoid of HPV infection (Crook *et al.*, 1991; Scheffner *et al.*, 1991; Iwasaka *et al.*, 1993). In the present study, although p53 mutations were more frequent in HPV-negative tumours (22.2% *vs* 50.0%), there was no statistically significant difference between HPV-positive and -negative carcinomas ($P = 0.1946$). This divergence may be due to different aetiological contribution of carcinogenic factors in pathogenesis of these two carcinomas.

As mentioned above, inactivation of p53 by binding to E6 oncoprotein and by missense mutations are two distinct pathways. The mechanism leading to loss of tumour-suppressor activity by binding to the HPV E6 transforming proteins differs considerably from that due to p53 gene mutations or allelic losses. Tumours resulting from HPV infection and consequently E6 expression may contain only wild-type p53 gene (Crook *et al.*, 1991; Howley, 1991; Scheffner *et al.*, 1991; Iwasaka *et al.*, 1993). On the other hand, mutations or allelic losses in the p53 gene largely derive from exposure to exogenous carcinogens (Harris, 1991; Hollstein *et al.*, 1991*a*; Chang *et al.*, 1993*a*,*b*). These two factors may act independently on the cells, but may sometimes act on the same cell and cooperate with each other. The presence of both HPV DNA sequences and p53 mutations in the same tumours in the present study provides direct evidence for such a cooperation.

Although HPV infections have been closely associated with the development of a primary cervical carcinomas, it seems likely that the initial HPV-induced lesions represent a premalignant stage and that additional initiating factors are required for a fully malignant transformation (Syrjänen *et al.*, 1987; zur Hausen, 1991). This is also seen in HPV-immortalised primary human epithelial cells which are initially non-transformed but acquire a tumorigenic phenotype by subsequent infection with an activated *ras* oncogene (DiPaolo *et al.*, 1989; Hurlin *et al.*, 1991) or treatment with a very low amount of a carcinogenic agent, e.g. nitrosamines (Garrett *et al.*, 1993), benzo[*a*]pyrene and methanesulphonic acid ethyl ester (Li *et al.*, 1992). The results of the present study provide direct evidence to support the hypothesis that certain HPV genomes are essential but not sufficient for progression to malignancies and that synergistic actions with other carcinogenic agents are required (zur Hausen, 1991). Exposure to environmental mutagens or carcinogens causes mutation or loss of one wild-type allele of the p53 or *RB* gene, leading to a reduced concentration of the wild-type p53. Cells with this genetic damage may acquire a selective growth advantage, but still show benign phenotype. If the cells are simultaneously infected with HPV, the reduced level of the wild-type p53 could certainly enhance the ability of E6/E7 proteins to complex with all p53 or RB proteins. However, in patients developing a tumour without HPV infection, the remaining p53 allele must be inactivated through either point mutations or allelic losses.

As compared with the epithelium of the uterine cervix, the oesophageal mucosa is continuously exposed to higher levels of a large number of environmental carcinogens (Chang *et al.*, 1992*a*), many of which are known to elicit DNA base substitutions and cause gene mutations either in bacteria and mammalian cells *in vitro* or in experimental animals *in vivo* (Harris, 1991; Holstein *et al.*, 1991*a*). These factors may act synergistically with HPV, leading to the development of carcinomas. This is in agreement with the increasing number of reports on the high percentage of the gene mutations or allelic losses of p53, *RB* and other tumour-suppressor genes in oesophageal carcinomas (Hollstein *et al.*, 1990; Bennett *et al.*, 1991, 1992; Boynton *et al.*, 1991; Casson *et al.*, 1991, Hollstein *et al.*, 1991*b*; Meltzer *et al.*, 1991; Greenwald *et al.*, 1992; Huang *et al.*, 1992, 1993). Indeed, base substitutions are particularly frequent (60%) in patients who are consumers of both tobacco and alcoholic beverages (Hollstein *et al.*, 1991*a*), the two most widespread risk factors for oesophageal cancer. The frequent mutations of the p53 gene in both HPV-positive and -negative oesophageal carcinomas indicate that exposure to environmental carcinogens represents the predominant aetiological factor in the development of oesophageal cancer, and infection with HPV may be one of the promoting agents in a multistep process of oesophageal carcinogenesis.

In conclusion, this study confirms and extends previous reports indicating an important role for p53 gene in eosophageal carcinogenesis. In addition to the high frequency of p53 mutations in HPV-negative oesophageal cancers, genetic alterations in the p53 gene were also common in HPV-positive carcinomas. This suggests an intriguing possibility that p53 mutations and HPV E6 oncoprotein may cooperate in the pathogenesis of some oesophageal carcinomas.

This study was supported by a research grant from the Savo Cancer Fund (F.C.) and in part by a research grant from the Finnish Cancer Society, a joint research grant from Fabriques de Tabac Reunies S.A., and British-American Tobacco Company Ltd, and a research grant from the Medical Research Council of the Academy of Finland. The skilful technical assistance of Mrs Kaarina Hoffrén, Mrs Aija Korkalainen, Mrs Helena Kemiläinen and Mrs Marja Nykänen is gratefully acknowledged. The authors extend their special thanks to Professor Dr Lutz Gissmann, Professor Dr Harald zur Hausen, DKFZ, Heidelberg, Germany and to Professor Gerard Orth, Pasteur Institute, Paris, France, for placing the HPV DNA probes at our disposal.

**References**

BENAMOUZIG, R., PIGOT, F., QUIROGA, G., DALIDIRE, P., CHAUSSADE, S., CATALAN, F. & COUTURIER, D. (1992). Human papillomavirus infection in esophageal squamous-cell carcinoma in Western countries. *Int. J. Cancer*, **50**, 549-552.

BENNETT, W.P., HOLLSTEIN, M.C., HE, A., ZHU, S.M., RESAU, J.H., TRUMP, B.F., METCALF, R.A., WELSH, J.A., MIDGLEY, C., LANE, D.P. & HARRIS, C.C. (1991). Archival analysis of p53 genetic and protein alterations in Chinese esophageal cancer. *Oncogene*, **6**, 1779-1784.

BENNETT, W.P., HOLLSTEIN, M.C., METCALF, R.A., WELSH, J.A., HE, A., ZHU, S.M., KUSTERS, I., RESAU, J.H., TRUMP, B.F., LANE, D.P. & HARRIS, C.C. (1992). p53 mutation and protein accumulation during multistage human esophageal carcinogenesis. *Cancer Res.*, **52**, 6092-6097.

BLOUNT, P.L., RAMEL, S., RASKIND, W.H., HAGGITT, R.C., SANCHEZ, C.A., DEAN, P.J., RABINOVITCH, P.S. & REID, B.J. (1991). 17p allelic deletions and p53 protein overexpression in Barrett's adenocarcinoma. *Cancer Res.*, **51**, 5482-5486.

BOYNTON, R.F., HUANG, Y., BLOUNT, P.L., REID, B.J., RASKIND, W.H., HAGGITT, R.C., NEWKIRK, C., RESAU, J.H., YIN, J., McDANIEL, T. & MELTZER, S.J. (1991). Frequent loss of heterozyosity at the retinoblastoma locus in human esophageal cancers. *Cancer Res.*, **51**, 5766-5769.

BUCHMAN, V.L., CHUMAKOV, P.M., NINKINA, N.N., SAMARINA, O.P. & GEORGIEV, G.P. (1988). A variation in the structure of the protein-coding region of the human p53 gene. *Gene*, **70**, 245-252.

CASSON, A.G., MUKHOPADHYAY, T., CLEARY, K.R., RO, J.Y., LEVIN, B. & ROTH, J.A. (1991). p53 gene mutations in Barrett's epithelium and esophageal cancer. *Cancer Res.*, **51**, 4495-4499.

CHANG, F., SYRJÄNEN, S., SHEN, Q., JI, H. & SYRJÄNEN, K. (1990). Human papillomavirus (HPV) DNA in esophageal precancer lesions and squamous cell carcinomas from China. *Int. J. Cancer*, **45**, 21-25.

CHANG, F., SYRJÄNEN, S., WANG, L. & SYRJÄNEN, K. (1992*a*). Infectious agents in the etiology of esophageal cancer. *Gastroenterology*, **103**, 1136-1148.

CHANG, F., SYRJÄNEN, S., SHEN, Q., WANG, L., WANG, D. & SYRJÄNEN, K. (1992*b*). Human papillomavirus (HPV) involvement in esophageal precancer lesions and squamous cell carcinomas as evidenced by microscopy and different DNA-techniques. *Scand. J. Gastroenterol.*, **27**, 553-563.

CHANG, F., SYRJÄNEN, S., KURVINEN, K. & SYRJÄNEN, K. (1993*a*). The p53 tumor suppressor gene as a common cellular target in human carcinogenesis. *Am. J. Gastroenterol.*, **88**, 174-186.

CHANG, F, SYRJÄNEN, S., TERVAHAUTA, A. & SYRJÄNEN, K. (1993*b*). Tumorigenesis associated with the p53 tumour suppressor gene. *Br. J. Cancer*, **68**, 653-661.

CHANG, F., SYRJÄNEN, S. & SYRJÄNEN, K. (1993*c*). Demonstration of human papillomavirus (HPV) type 30 in esophageal squamous-cell carcinomas by *in situ* hybridization. *Int. J. Cancer*, **55**, 171-173.

CHANG, F., SYRJÄNEN, S., SHEN, Q., WANG, L. & SYRJÄNEN, K. (1993*d*). Screening for human papillomavirus (HPV) infections in esophageal squamous cell carcinomas by in situ hybridization. *Cancer*, **72**, 2525-2530.

CROOK, T., WREDE, D. & VOUSDEN, K.H. (1991). p53 point mutation in HPV negative human cervical carcinoma cell lines. *Oncogene*, **6**, 873-875.

DIPAOLO, J.A., WOODWORTH, C.D., POPESCU, N.C., NOTARIO, V. & DONIGER, J. (1989). Introduction of human cervical squamous cell carcinoma by sequential transfection with human papillomavirus 16 DNA and viral Harvey ras. *Oncogene*, **4**, 395-399.

FREBOURG, T. & FRIEND, S.H. (1992). Cancer risks from germline p53 mutations. *J. Clin. Invest.*, **90**, 1637-1641.

GARRETT, L.R., PEREZ-REYES, N., SMITH, P.P. & McDOUGALL, J.K. (1993). Interaction of HPV-18 and nitrosomethylurea in the induction of squamous cell carcinoma. *Carcinogenesis*, **14**, 329-332.

GREENWALD, B.D., HUANG, Y., BAUM, R. & MELTZER, S.J. (1992). Barrett's carcinoma in a 25-year-old man with point mutation of the p53 tumor suppressor gene. *Int. J. Oncol.*, **1**, 271-275.

HARRIS, C.C. (1991). Chemical and physical carcinogenesis: advances and perspectives for the 1990s. *Cancer Res.*, **51** (Suppl)., 5023s-5044s.

HAYASHI, K. (1992). PCR-SSCP: a method for detection of mutations. *GATA*, **9**, 73-79.

HOLLSTEIN, M.C., METCALF, R.A., WELSH, J.A., MONTESANO, R. & HARRIS, C.C. (1990). Frequent mutation of the p53 gene in human esophageal cancer. *Proc. Natl Acad. Sci. USA*, **87**, 9958-9961.

HOLLSTEIN, M., SIDRANSKY, D., VOGELSTEIN, B. & HARRIS, C.C. (1991*a*). p53 mutations in human cancers. *Science*, **253**, 49-53.

HOLLSTEIN, M.C., PERI, L., MANDARD, A.M., WELSH, J.A., MONTESANO, R., METCALF, R.A., BAK, M. & HARRIS, C.C. (1991*b*). Genetic analysis of human esophageal tumors from two high incidence geographic areas: frequent p53 base substitutions and absence of *ras* mutations. *Cancer Res.*, **51**, 4102-4106.

HOWLEY, P.M. (1991). Role of the human papillomaviruses in human cancer. *Cancer Res.*, **51**, 5019-5022.

HUANG, Y., BOYNTON, R.F., BLOUNT, P.L., SILVERSTEIN, R.J., YIN, J., TONG, Y., McDANIEL, T.K., NEWKIRK, C., RESAU, J.H,. SRIDHARA, R., REID, B.J. & MELTZER, S.J. (1992). Loss of heterozygosity involves multiple tumor suppressor genes in human esophageal cancers. *Cancer Res.*, **52**, 6525-6530.

HUANG, Y., MELTZER, S.J., YIN, J., TONG, Y., CHANG, E.H., SRIVASTAVA, S., McDANIEL, T., BOYNTON, R.F. & ZOU, Z.Q. (1993). Altered messenger RNA and unique mutational profiles of p53 and Rb in human esophageal carcinomas. *Cancer Res.*, **53**, 1889-1894.

HURLIN, P.J., KAUR, P., SMITH, P.P., PEREZ-REYES, N., LANTON, R.A. & McDOUGALL, J.K. (1991). Progression of human papillomavirus type 18-immortalized human keratinocytes to a malignant phenotype. *Proc. Natl Acad. Sci. USA*, **88**, 570-574.

IWASAKA, T., OH-UCHIDA, M., MATSUO, N,. YOKOYAMA, M, FUKUDA, K., HARA, K., FUKUYAMA, K., HORI, K. & SUGIMORI, H. (1993). Correlation between HPV positivity and state of the p53 gene in cervical carcinoma cell lines. *Gynecol. Oncol.*, **48**, 104-109.

KULSKI, J., DEMETER, T., STERRET, G.F. & SHILKIN, K.B. (1986). Human papillomavirus DNA in esophageal carcinoma. *Lancet*, **ii**, 683-684.

LANE, D.P. (1992). p53, guardian of the genome. *Nature*, **358**, 15-16.

LEVINE, A.J. (1990). The p53 protein and its interactions with the oncogene products of the small DNA tumor viruses. *Virology*, **177**, 419-426.

LEVINE, A.J., MOMAND, J. & FINLAY, C.A. (1991). The p53 tumour suppressor gene. *Nature*, **351**, 453-456.

LI, S.-L., KIM, M.S., CHERRICK, H.M., DONIGER, J. & PARK, N.-H. (1992). Sequential combined tumorigenic effect of HPV-16 and chemical carcinogens. *Carcinogenesis*, **13**, 1981-1987.

LU, J.B., YANG, W.X., LIU, J.M., LI, Y.S. & QIN, Y.M. (1985). Trends in morbidity and mortality for oesophageal cancer in Linxian country. *Int. J. Cancer*, **36**, 643-645.

MELTZER, S.J., YIN, J., HUANG, Y., McDANIEL, T.K., NEWKIRK, C., ISERI, O., VOGELSTEIN, B. & RESAU, J.H. (1991). Reduction to homozygosity involving p53 in esophageal cancers demonstrated by the polymerase chain reaction. *Proc. Natl Acad. Sci. USA*, **88**, 4976-4980.

MILLER, C., MOHANDAS, T., WOLF, D., PROKOCIMER, M., ROTTER, V. & KOEFFLER, P.H. (1986). Human p53 localized to short arm of chromosome 17. *Nature*, **319**, 783-784.

ORITA, M., SUZUKI, Y., SEKIYA, T. & HAYASHI, K. (1989). Rapid and sensitive detection of point mutations and DNA polymorphisms using the polymerase chain reaction. *Genomics*, **5**, 874-879.

SARNOW, P., HO, Y.S., WILLIAMS, J. & LEVINE, A.J. (1982). Adenovirus E1b-58kd tumor antigen and SV40 large tumor antigen are physically associated with the same 54kd cellular protein in transformed cells. *Cell*, **28**, 387-394.

SCHEFFNER, M., MÜNGER, K., BYRNE, J.C. & HOWELY, P.M. (1991). The state of the p53 and retinoblastoma genes in human cervical cell lines. *Proc. Natl Acad. Sci. USA*, **88**, 5523-5527.

SCHMEIG, F.I. & SIMMONS, D.T. (1988). Characterization of the *in vitro* interaction between SV40 T antigen and p53: mapping of the p53 binding site. *Virology*, **164**, 132-140.

SYRJÄNEN, K., GISSMANN, L. & KOSS, L.G. (1987). *Papillomaviruses and Human Disease*. Springer: Heidelberg.

SYRJÄNEN, S., PARTANEN, P., MÄNTYJÄRVI, R. & SYRJÄNEN, K. (1988). Sensitivity of in situ hybridization techniques using biotin and $^{35}$S-labeled human papillomavirus (HPV) DNA probes. *J. Virol. Methods*, **19**, 225–238.

TOH., Y., KUWANO, H., TANAKA, S., BABA, K., MATSUDA, H., SUGIMACHI, K. & MORI, R. (1992). Detection of human papillomavirus DNA in esophageal carcinoma in Japan by polymerase chain reaction. *Cancer*, **70**, 2234–2238.

VOGELSTEIN, B. & KINZLER, K.W. (1992). p53 function and dysfunction. *Cell*, **70**, 523–526.

WAGATA, T., ISHIZAKI, K., IMAMURA, M., SHIMADA, Y., IKENAGA, M. & TOBE, T. (1991). Deletion of 17p and amplification of the *int*-2 gene in esophageal carcinomas. *Cancer Res.*, **51**, 2113–2117.

WERNESS, B.A., LEVINE, A.J. & HOWLEY, P.M. (1990). The E6 proteins encoded by human papillomavirus types 16 and 18 can complex p53 *in vitro*. *Science*, **248**, 76–79.

WILLIAMSON, A.I., JASKIESICZ, K. & GUNNING, A. (1991). The detection of human papillomavirus in oesophageal lesions. *Anticancer Res.*, **11**, 263–266.

WINKLER, B., CAPO, V., REUMANN, W., AVERILL, M.A., LAPORT, R., REILLY, S., GREEN, P.M.R., RICHART, R.A. & CRUM, C.P. (1985). Human papillomavirus infection of the esophagus: a clinicopathologic study with demonsrations of papillomavirus antigen by the immunoperoxidase technique. *Cancer*, **55**, 149–155.

ZUR HAUSEN, H. (1991). Viruses in human cancers. *Science*, **254**, 1167–1173.